# Follow-up study of airway microbiota in children with persistent wheezing

**DOI:** 10.1186/s12931-021-01806-9

**Published:** 2021-07-27

**Authors:** Lei Wu, Chencong Shen, Yuanling Chen, Xin Yang, Xiaofei Luo, Chengcheng Hang, Lingling Yan, Xuefeng Xu

**Affiliations:** Department of Rheumatology Immunology & Allergy, and Pulmonary Medicine, The Children’s Hospital, Zhejiang University School of Medicine, National Clinical Research Center for Child Health, Hangzhou, 310003 People’s Republic of China

**Keywords:** Airway, Children, Microbiota, Persistent wheezing

## Abstract

**Background:**

Increasing evidence revealed that airway microbial dysbiosis was associated with increased risk of asthma, or persistent wheezing (PW). However, the role of lung microbiota in PW or wheezing recurrence remains poorly understood.

**Methods:**

In this prospective observational study, we performed a longitudinal 16S rRNA-based microbiome survey on bronchoalveolar lavage (BAL) samples collected from 35 infants with PW and 28 age-matched infants (control group). A 2-year follow-up study on these PW patients was conducted. The compositions of lower airway microbiota were analyzed at the phylum and genus levels.

**Results:**

Our study showed a clear difference in lower airway microbiota between PW children and the control group. Children with PW had a higher abundance of *Elizabethkingia* and *Rothia*, and lower abundance of *Fusobacterium* compared with the control group. At the end of the 2-year follow-up, 20 children with PW (57.1%) experienced at least one episode of wheezing, and 15 (42.9%) did not suffer from wheezing episodes. Furthermore, PW children with recurrence also had increased abundances of *Elizabethkingia* and *Rothia* relative to those who had no recurrence. Additionally, wheezing history, different gender, and caesarean section demonstrated a greater impact in airway microbiota compositions.

**Conclusion:**

This study suggests that the alterations of lower airway microbiota could be strongly associated with the development of wheezing, and early airway microbial changes could also be associated with wheezing recurrence later in life.

## Introduction

Pediatric asthma is a common chronic disease of childhood and a major public health problem. Furthermore, asthma frequently begins in early childhood, and in up to half of asthma people, symptoms commence during childhood. Especially for children younger than 24 months, they usually manifest as repeated wheezing. Some infants will develop persistent or recurrent wheezing, which is often severe [1]. There is evidence that infantile wheezing or persistent wheezing (PW) is strongly associated with the development of asthma later in life [2]. However, the underlying mechanisms between them are still poorly understood. A number of biologically plausible mechanisms have suggested the effect of environmental changes early in life on the subsequent development of asthma, including cytokine response, developmental origins of adult disease, and microbial exposure [3–6].

The advances of culture-independent molecular technique of Next-Generation Sequencing (NGS) have confirmed the large number of microbiological organisms in the lungs of healthy individuals and patients with asthma [7]. The interactions between host and microbiota not only influence immune system development, but also play a significant role in regulating various inflammatory and metabolic pathways in the development of asthma [8, 9]. Furthermore, the airway microbial dysbiosis is associated with increased risk of asthma and asthma features [10, 11].

Currently, most studies on the role of airway microbiota in pediatric asthma or wheezing mainly focused on upper airway [12–15]. The data from lower airway samples might better reflect the connection between airway microbiota and the development of wheezing. A certain airway microbiota profile could be related with PW or wheezing recurrence in PW children. Here, we performed a 16S rRNA-based microbiome survey on bronchoalveolar lavage (BAL) samples collected from children with PW, aiming to explore their airway microbiota compositions. At the same time, we also conducted a follow-up study on these PW patients, observing whether the recurrence of wheezing is related to the specific airway microbiota spectrum.

## Methods

### Study design and BAL collection

In this prospective observational cohort study, hospitalized children with PW were recruited from Children’s Hospital of Zhejiang University School of Medicine. This study was approved by the Ethic Review Board of Children’s Hospital, Zhejiang University School of Medicine (2018-IRB-069). Informed consent was obtained for all the subjects who are under 18 from a parent and/or legal guardian. Diagnosis of persistent wheezing was based on persistent episodes of infantile wheezing [1]. In brief, inclusion criteria were as follow: (1) younger than 24 months; (2) duration of wheezing episode more than one month despite treatment with recommended first-line therapies of bronchodilators, inhaled corticosteroids, or systemic corticosteroids. All the included children were followed up to assess the recurrence of wheeze within 24 months of recruitment. The development of wheezing was confirmed by pediatrician, mainly based on wheezing symptoms and signs. At the same time, 28 age-matched infants underwent bronchoscopy due to bronchial granulation caused by foreign body aspiration, and these infants did not use any antibiotics and labeled as control group. Detailed clinical characteristics between children with PW and control subjects were listed in Table 1.

**Table 1:** Characteristics of children with persistent wheezing and control children

	Persistent wheezing (n = 35)	Control (n = 28)	*P* value
Age (month)	13.46 ± 3.43	15.64 ± 5.31	0.066
Gender (F/M)	15/20	12/16	1.0
Cesarean section	12	18	0.024*
WBC counts (× 10^9^/L)	10.68 ± 3.18	10.53 ± 2.92	0.85
Neutrophils (%)	38.46 ± 16.01	33.11 ± 8.57	0.096
Eosinophils (%)	1.53 ± 1.77	2. 12 ± 1.71	0.187
Hgb (g/L)	119.63 ± 10.71	118.82 ± 10.14	0.762
PLT (× 10^9^/L)	396.26 ± 119.92	375.11 ± 136.21	0.515
CRP (0–8 mg/L)	3.85 ± 8.00	1.85 ± 2.33	0.206
ALT	28.94 ± 32.88	18.21 ± 6.92	0.068
AST	53.03 ± 29.88	47.46 ± 23.09	0.421
LDH	404.97 ± 175.41	428.96 ± 247.51	0.654

*WBC* white blood cells, *Hgb* hemoglobin, *PLT* blood platelet, *CRP* C reactive protein, *ALT* alanine aminotransferase, *AST* aspartate aminotransferase, *LDH* lactate dehydrogenase

^*^ P < 0.05, ** P < 0.01

Bronchoalveolar lavage fluid (BAL) collections were based on our previous study [16]. Bronchoscopy was transnasally performed using a flexible video-bronchoscope (XP260F, Olympus, Tokyo, Japan) following general anesthesia. To reduce irritation to the throat, local throat spray with lidocaine was performed. BAL was done by instillation of sterile saline (1 ml/kg, max 20 ml), and the collected BAL was centrifuged at 14000×*g* for 10 min. The pellets were re-suspended in 500 µL sterile PBS and stored at – 80 ℃ until DNA extraction.

### DNA extraction and 16S rRNA gene sequencing

DNA extraction and 16S rRNA gene sequencing methods were based on our previous study [16]. Briefly, total genomic DNA was extracted using a commercial kit according to the manufacturer’s instruction (DP382, Tiangen, Beijing, China). The extracted DNA were quantified with NanoDrop ND-1000 spectrophotometer (NanoDrop Technologies, Wilmington, DE, USA) and stored at – 80 ℃ until amplification. An aliquot of DNA from each BAL sample was used as the template for amplifications. To characterize the bacterial microbiota, the V3 to V4 regions of 16S rRNA gene were amplified (Forward primer 5′-CCTACGGGNGGCWGCAG-3′ and Reverse primer 5′-GACTACHVGGGTATCTAATCC-3′), barcoded, and sequenced on the Illumina Miseq sequencing platform (Illumina, San Diego, USA).

### Data processing and statistical analysis

Quality filtering, denoising, and chimera removal were performed using the open-source software package, Quantitative Insights Into Microbial Ecology (QIIME, 1.9.13) platform [17]. Operational taxonomic units (OTUs) were defined using an identity cutoff value of 97% as this generally approximates the difference in 16S sequences between bacterial species [18]. Taxa with the relative abundances > 0.1% was included in hierarchical clustering. The alpha-diversity estimates describe the number of species in a single sample, and it was calculated within QIIME using the Chao1 and Shannon indexes. Taxonomy was assigned using the rdp-classifier-2.11 in QIIME (http://www.qiime.org) trimmed to sequences with complete taxonomic path. The rarefied sequence depth from 10 to the maximum value was set, and thirty points among them were determined. Rarefied samples were used to calculate alpha and beta diversity. The core microbiota was identified at the phylum and genus level.

All the statistical analysis and graphics were performed with R statistical software packages (R version 3.4.3). Statistical analysis was performed using descriptive statistics. The continuous variables between groups were compared by Student’s t-test or Mann–Whitney U test. Spearman rank correlations were used to demonstrate the association between various bacterial genera. For categorical variables, Pearson's chi-squared test was applied. A *P* value of less than 0.05 was considered statistically significant in all the analysis.

## Results

### Characteristics of included children

During this study period, 35 children with PW were enrolled, and completed the follow-up (Table 1). Of them, 20 children (57.1%) experienced at least one episode of wheezing (recurrence) at the end of the 2-year follow-up, and 15 (42.9%) did not suffer from wheezing episodes (no recurrence). Compared with PW children without recurrence, PW children with recurrence had frequently a history of wheezing (46.7% vs 95%, *P* = 0.002, Table 2). Although antibiotics administration had the impact on the airway microbiota, there were no significant differences observed in β-lactam and macrolides uses before hospitalization between the two groups.

**Table 2:** Characteristics of persistent wheezing children with and without recurrence

	Patients without recurrence (n = 15)	Patients with recurrence (n = 20)	P value
Age (m)	13.47 ± 3.83	13.45 ± 3.20	0.99
Gender (F/M)	7/8	13/7	0.32
Cesarean section (n)	6	6	0.72
Wheeze history (n)	7	19	0.002**
Follow-up time (m)	17.27 ± 6.16 (10–24)	17.60 ± 5.00 (10–24)	0.86
WBC counts (× 10^9^/L)	11.27 ± 3.87	10.23 ± 2.56	0.34
Neutrophils %	37.74 ± 19.90	39.00 ± 12.89	0.82
Eosinophils %	1.34 ± 1.87	1.68 ± 1.73	0.59
Hgb	120.13 ± 10.72	119.25 ± 10.96	0.81
PLT	386.47 ± 90.91	403.60 ± 139.69	0.68
CRP (0 – 8 mg/L)	3.92 ± 10.60	3.80 ± 5.63	0.96
PCT (< 0.5 ng/L)	0.06 ± 0.04	0.08 ± 0.07	0.21
ALT	35 ± 45	24 ± 19	0.35
AST	61.13 ± 36.87	46.95 ± 22.47	0.17
LDH	454.60 ± 207.40	367.75 ± 141.29	0.15
IgG	6.35 ± 1.45	7.25 ± 1.45	0.08
IgA	0.36 ± 0.27	0.53 ± 0.23	0.06
IgE	106.32 ± 192.22	126.28 ± 226.08	0.79

*WBC* white blood cells, *Hgb* hemoglobin, *PLT* blood platelet, *CRP* C reactive protein, *PCT* procalcitonin, ALT alanine aminotransferase, *AST* aspartate aminotransferase, *LDH* lactate dehydrogenase, *Ig* Immunoglobulin

^*^ P < 0.05, ** P < 0.01

### Airway microbiota composition of children with PW

A total of 2,179,174 high-quality tags were generated, averaging 34,024 and 35,298 for the persistent wheezing and control groups, respectively. The average OTU numbers in the PW and control groups were 254 and 296 (*P* = 0.17), respectively. There were also no significant differences in the bacterial alpha diversity between the two groups using Chao1 (379 ± 203 vs 484 ± 248, *P* = 0.07) and Shannon indexes (3.7 ± 1.0 vs 4.0 ± 0.9, *P* = 0.35).

**Fig. 1:**
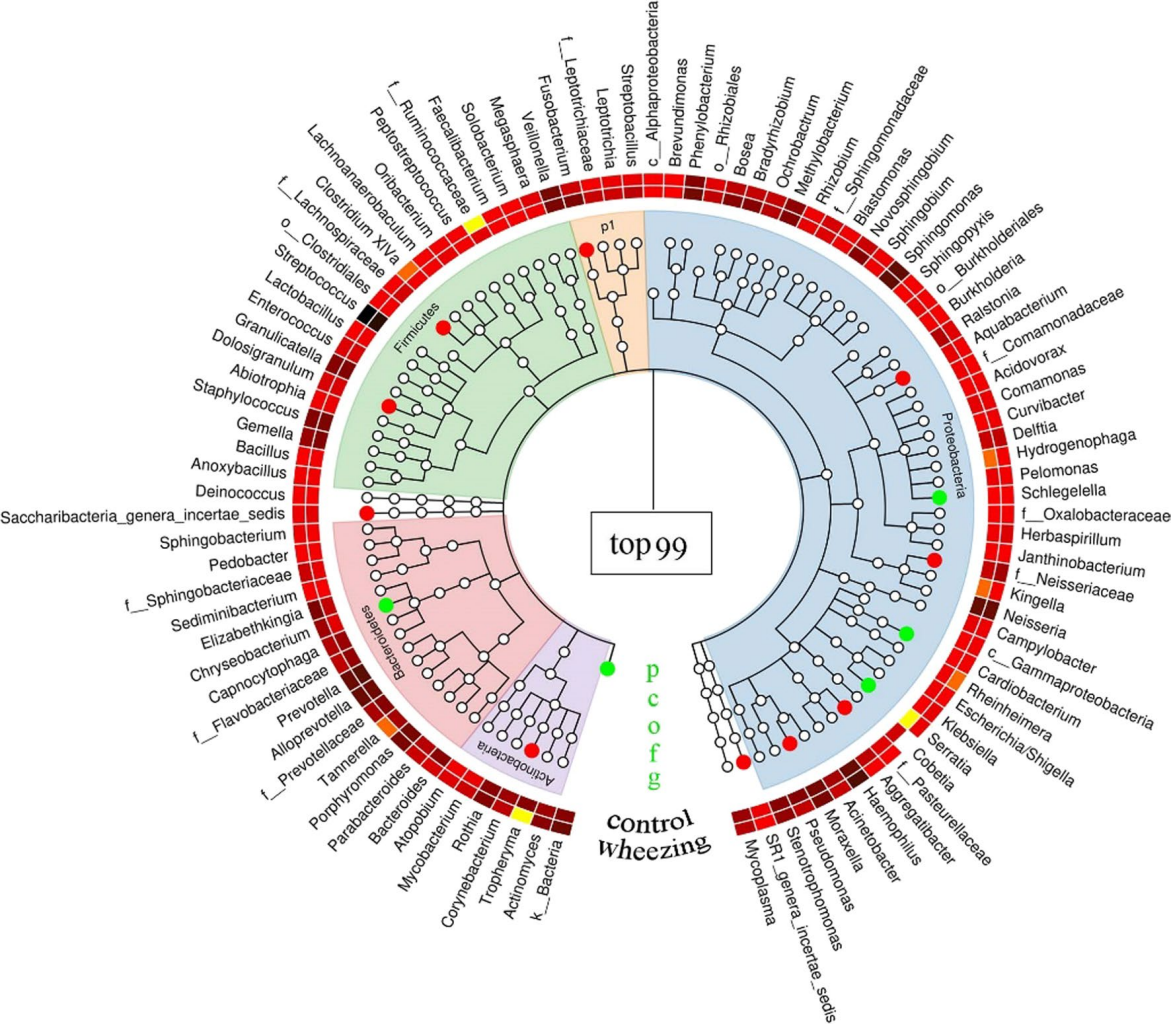
A heatmap of the airway microbiota composition at levels of phylum (P), class (C), order (O), family (F), and genus (G). The innermost layer is the taxonomic tree labeled with background color, and the outermost layer is the annotated genus. The red globules indicated that there was significant difference between children with persistent wheezing and control group. The middle layer with red and yellow was a heatmap of average genus abundance, the darker the color, the higher the abundance

**Fig. 2:**
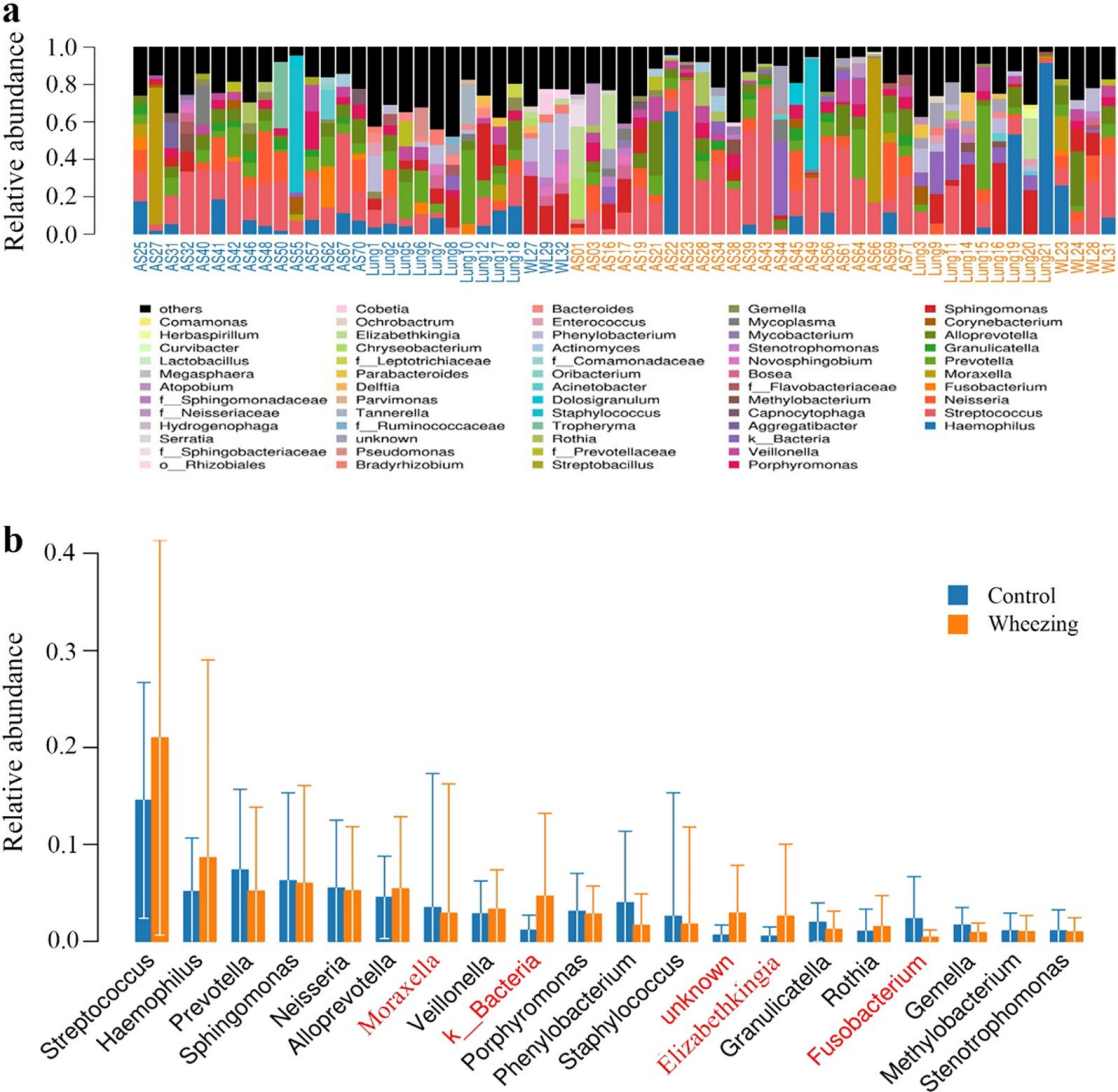
Relative abundance plot of airway microbiota from children with persistent wheezing and control group. A showed a proportional abundance in each BAL sample at the genus level. B showed a relative abundance at the genus level between children with persistent wheezing and control group. The red-labeled bacterial genera represent significant differences (*P* < 0.05) between the two groups

A heatmap showed the airway microbiota composition at levels of phylum, class, order, family, and genus (Fig. 1). Overall, four dominant bacterial phyla were detected: *Proteobacteria* (35.5% vs 39.6%, *P* = 0.53), *Firmicutes* (30.3% vs 26.7%, *P* = 0.52), *Bacteroidetes* (20.6% vs 21.9%, *P* = 0.75), and *Actinobacteria* (4.2% vs 4.6%, *P* = 0.80) from children with PW and control group, respectively. Figure 2 showed a relative abundance of bacteria at the genus levels in each child. At the genus level, children with PW had a higher abundance of *Elizabethkingia* (2.6% vs 0.5%, *P* = 0.011) and *Rothia* (1.5% vs 1.0%, *P* = 0.27), and lower abundances of *Moraxella* (2.9% vs 3.5%, p = 0.04) and *Fusobacterium* (0.4% vs 2.3%, *P* = 0.004) relative to control group.

### Effects of gender, delivery mode, and wheezing history on airway microbiota among children with PW

**Fig. 3:**
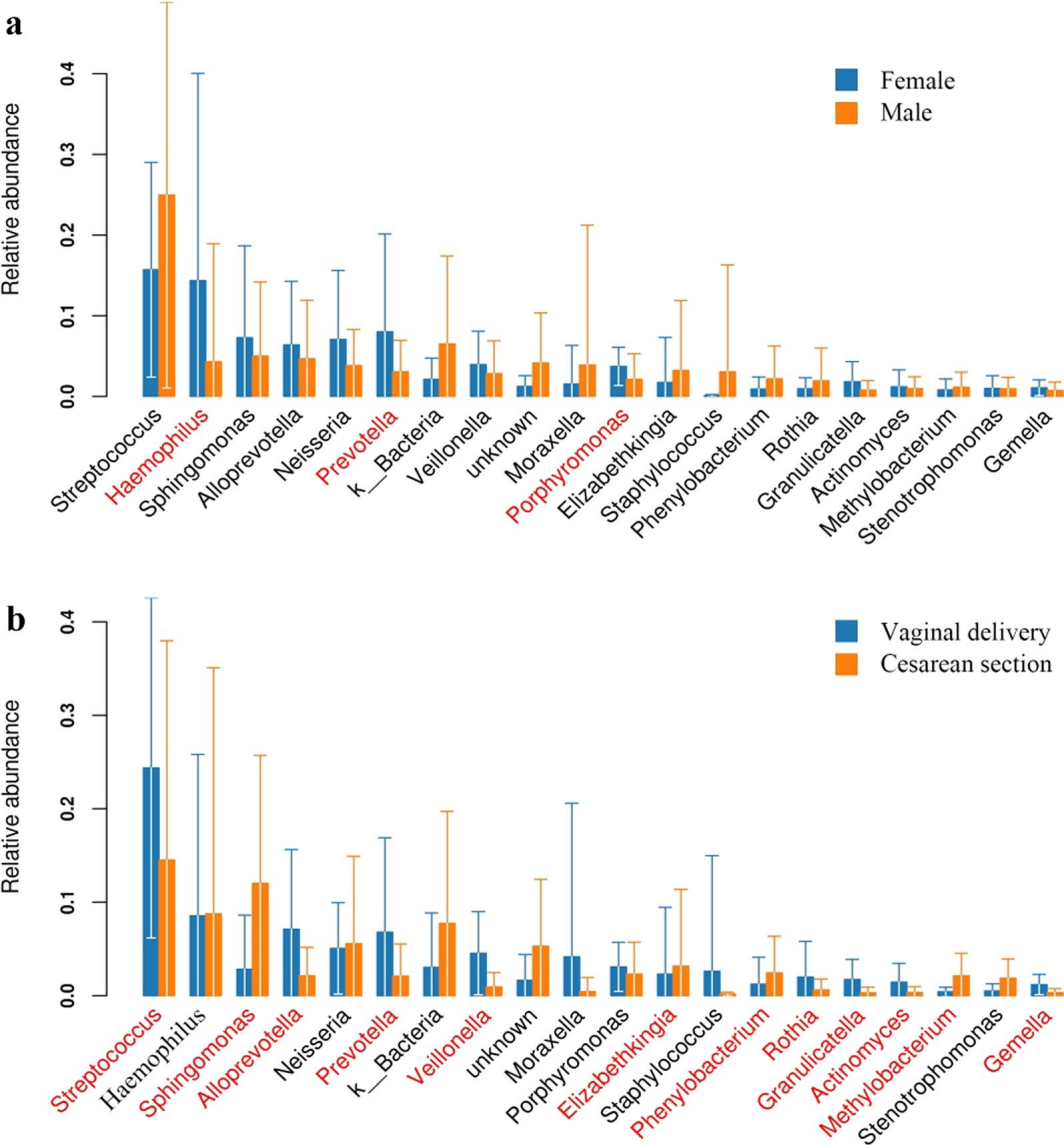
Relative abundance plot of bacterial genus level from children with persistent wheezing. Bar graph A showed a difference between female and male groups, and B showed a difference between vaginal delivery and cesarean section groups. The red-labeled bacterial genera represent significant differences (*P* < 0.05) between two groups

**Fig. 4:**
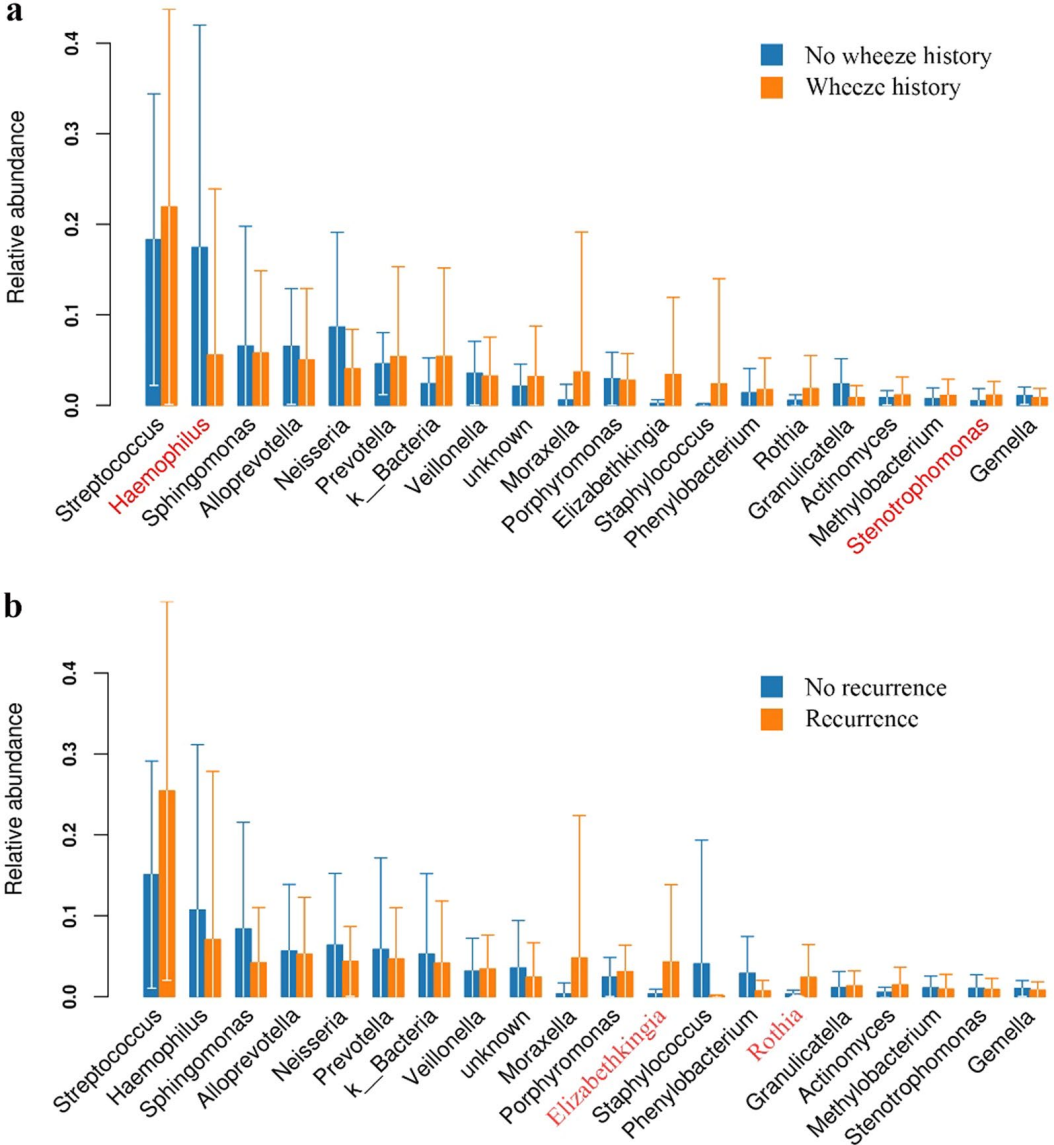
Relative abundance plot of bacterial genus level from children with persistent wheezing. Bar graph A showed a difference between children with and without wheeze history, and B showed a difference between children with and without recurrence. The red-labeled bacterial genera represent significant differences (*P* < 0.05) between two groups

No significant differences were observed in four dominant bacterial phyla proportions between boys and girls, vaginal delivery and cesarean section groups, and children with or without wheezing history. However, at the genus levels, boys with PW had lower abundances of *Haemophilus, Prevotella*, and *Prophyromonas* (*P* < 0.05, respectively) compared with girls with PW (Fig. 3A). Relative to PW children with vaginal delivery, PW children with cesarean section had lower abundances of *Streptococcus*, *Alloprevotella*, *Prevotella*, and *Rothia* (P < 0.05, respectively), and higher abundances of *Sphingomonas, Elizabethkingia,* and *Phenylobacterium* (P < 0.05, Fig. 3B). PW children with wheezing history had a higher abundance of *Elizabethkingia,* and a lower abundance of *Haemophilus* (P < 0.05) compared with those without wheezing history. Despite no statistical difference, an increased abundance of *Rothia* was observed in PW children with wheezing history (Fig. 4A).

### Airway microbiota changes between PW children with and without recurrence

To further understand the role of airway microbiota in the development of wheezing recurrence among children with PW, we performed a follow-up study. At the bacteria phylum level, no significant differences were observed with respect to the proportion of *Proteobacteria*, *Firmicutes*, *Bacteroidetes*, and *Actinobacteria* between children with and without recurrence. Notably at the genus level, PW children with recurrence had increased abundance of *Elizabethkingia* (4.3% vs 0.3%, *P* = 0.002) and *Rothia* (2.4% vs 0.3%, *P* = 0.004), indicating that a different airway microbiota profile might be associated with the recurrence of wheezing (Fig. 4B).

### Rank Correlation among the predominant genera in PW children

**Fig. 5:**
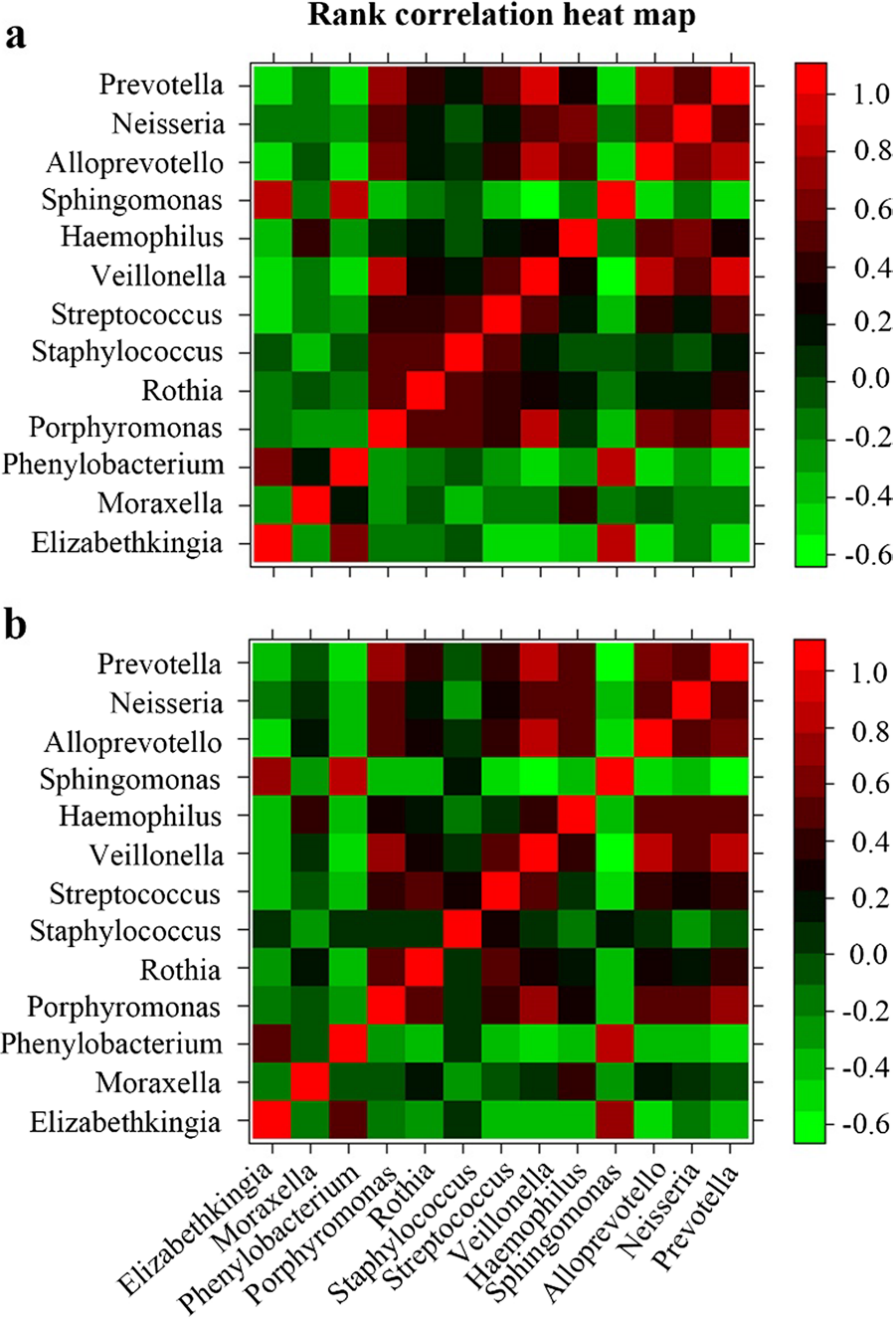
Rank correlation heatmap between bacterial genus abundances in children with persistent wheezing. A showed a correlation matrix of bacterial genus abundances in children with persistent wheezing (n = 35), and B showed a correlation matrix in children with recurrence (n = 20). Red represents a positive correlation, and green indicates a negative correlation. Noted significant correlations of *Elizabethkingia* with *Phenylobacterium*, *Streptococcus*, *Sphingomonas*, *Alloprevotella*, and *Prevotella, and Rothia* with *Porphyromonas*, *Staphylococcus*, and *Streptococcus*

Significant rank correlations between the predominant genera *Elizabethkingia, Phenylobacterium*, *Porphyromonas*, *Rothia*, *Streptococcus*, *Veillonella*, *Haemophilus*, *Sphingomonas, Alloprevotella*, *Neisseria*, and *Prevotella* were found among children with PW (Fig. 5A). The relative abundance of *Elizabethkingia* was positively correlated with abundances of *Phenylobacterium* (r = 0.532, *P* = 0.001) and *Sphingomonas* (r = 0.735, P < 0.001), negatively correlated with abundances of *Streptococcus* (r = − 0.398, *P* = 0.018), *Veillonella* (r = − 0.409, *P* = 0.015), *Alloprevotella* (r = − 0.448, *P* = 0.007), and *Prevotella* (r = − 0.429, *P* = 0.01). The relative abundance of *Rothia* was positively correlated with abundances of *Porphyromonas* (r = 0.519, *P* = 0.001), *Streptococcus* (r = 0.536, *P* = 0.001), and *Prevotella* (r = 0.433, *P* = 0.009), negatively correlated with abundances of *Sphingomonas* (r = − 0.411, *P* = 0.014).

Additionally, in PW children with recurrence, the genus *Elizabethkingia* also showed similar correlation trend with abundances of *Phenylobacterium*, *Sphingomonas*, *Streptococcus*, *Alloprevotella*, and *Prevotella*. Although the relative abundance of *Rothia* was positively correlated with abundances of *Porphyromonas* (r = 0.487, *P* = 0.029), and *staphylococcus* (r = 0.474, *P* = 0.035), *Rothia* indicated a weak correlation with *Streptococcus* (r = 0.442, *P* = 0.051, Fig. 5B).

## Discussion

This prospective study demonstrated a distinct characterization of airway microbiota in PW children younger than 24 months, and its associations with subsequent wheezing recurrence. We found that children with PW had higher abundances of *Elizabethkingia* and *Rothia*, and a lower abundance of *Fusobacterium*. Furthermore, during the follow-up period, PW children with recurrence also had increased abundances of *Elizabethkingia* and *Rothia* relative to those who had no recurrence.

Although we did not find significant difference at the phylum level between children with PW and control group, the different dominant genera compositions were observed between the two groups. A previous study revealed that early *Moraxella*-dominant nasal microbiota profile in infants aged 2 months was associated with an increased rate of acute respiratory infections during the first 2 years of life [14]. The dominant *Moraxella* species of nasal microbiotas in older children with asthma were associated with increased exacerbation risk and eosinophil activation [13]. This increased risk is likely to be correlated with significantly greater epithelial damage and inflammatory cytokine expression induced by *Moraxella* species [13]. The higher abundance of *Haemophilus* and *Moraxella* in airway microbiota might regulate airway inflammation during severe infantile bronchiolitis, potentially contributing to the development of subsequent wheezing recurrence in later childhood [3]. Moreover, dominant genera can vary with age. Older children had higher randomization abundance of *Staphylococcus*, while younger children had higher abundances of *Moraxella* and *Streptococcus* [4].

Different from the above studies, our study demonstrated that children with PW had higher abundances of *Elizabethkingia* and *Rothia*, and lower abundances of *Moraxella* and *Fusobacterium* in lower airway microbiota. This finding was similar to a previous study by Kazachkov et al*.* showing an enriched *Rothia* in lower airway microbiota from asthma children older than two years [19]. Members of the genus *Elizabethkingia* are gram-negative and nonmotile rods, and they can cause pneumonia, catheter-related bacteremia, neonatal meningitis, and even outbreaks [20, 21]. So far, no report has been published regarding association between *Elizabethkingia* and asthma or wheezing. It is likely that an increased abundance of *Elizabethkingia* might be involved in the pathogenesis of persistent wheezing. Nevertheless, whether overabundance of *Elizabethkingia* is the cause or consequence of persistent wheezing are not fully understood. Another increased trend of *Rothia* in lower airway microbiota was observed in PW children, although it was not statistically significant. Additionally, PW children with wheezing history also possessed higher abundances of *Elizabethkingia* and *Rothia.* These findings strongly suggested a close association between increased abundances of *Elizabethkingia* and *Rothia* and infantile wheezing. However, PW children showed a lower abundance of *Moraxella,* which could be associated with different age and sampling location. Although PW children with recurrence had a higher abundance of *Moraxella* compared with those without recurrence, there was no significant difference between the two groups. This difference still needs to be further investigated to confirm. On the other hand, we also found significant rank correlations between *Elizabethkingia* or *Rothia* and other predominant genera *Phenylobacterium*, *Porphyromonas*, *Streptococcus*, *Veillonella*, *Haemophilus*, *Sphingomonas, Alloprevotella*, *Neisseria*, and *Prevotella*. The rank correlations among the predominant genera indicated that specific microbiota profiles could be associated with the development or progression of infantile wheezing. However, the underlying mechanisms still need further investigation.

Notably in this study, different gender and delivery mode also illustrated a greater impact in airway microbiota compositions, revealing the complexity of the formation of airway flora. Although overall gut microbial community structure at age 2–4 years was not associated with preschool wheezing or future asthma development at age 6 [22], our study on lower airway microbiota demonstrated a correlation between them. Interestingly in the present 2-year follow-up, we found that PW children who had at least one episode of wheezing recurrence also had increased abundances of *Elizabethkingia* and *Rothia* relative to those who had no recurrence. It further indicated that there is a clear correlation between airway microbiota and wheezing recurrence. This early specific alterations in airway microbiota are likely to indicate an increased risk of wheezing recurrence. Nevertheless, whether airway microbial alteration is the cause or consequence of wheezing needs further investigation. On the other hand, lung microbiota might be affected by many external factors. Environmental factors such as bed dust could influence airway microbiota compositions among infants [23]. Maternal dietary intervention with long-chain fatty acids and vitamin D supplementation can affect the infant airways but not the infant fecal microbiota [24]. Additionally, other potential confounders including living areas and living conditions can also have a certain impact on airway microbiota. This will further complicate the airway microbiota research.

Currently, 16S rRNA gene amplicon analysis remains the standard approach for the cultivation independent investigation of microbiota, and the accuracy of these analyses depends mainly on primer selections [25]. The nine hypervariable regions (V1-V9) of 16S rRNA gene are frequently used for determining of the bacterial taxonomy in the diverse microbial populations [4, 14, 23]. In general, high-throughput short-read sequencing of the 16S rRNA gene amplicon based on the Illumina MiSeq platform specifically targets the V3-V4 hypervariable region [26]. This region provides ample information for taxonomic classification of microbial communities from specimens associated with human microbiome studies [27]. However, due to the limitations of the technology itself, we did not definitely detect the species. Although bacterial culturing was routinely performed for BAL specimens, we failed to find the pathogenic bacteria or the same bacteria as those detected by 16S rRNA sequencing method. This phenomenon may be associated with less microbial ratios or antibiotic use before hospitalization. Now, a new 16S full-length-based synthetic long-read sequencing technology can be a choice to read the whole variable regions of 16S rRNA gene to identify the microbial communities in metagenome studies [26].

The present study has some limitations that should be considered. First, our study mainly focused on bacterial abundance via 16S rRNA analysis, not involved in the investigations of virus and fungi. A comprehensive analysis of bacterial, viral, and fungal microbiota would contribute us to better understanding host-microbiota interaction. Second, the influences of many other factors that might induce airway dysbiosis, are difficult to exclude, including corticosteroid and antibiotic uses. Although antibiotics have a major role in airway microbiota composition, antibiotic use will be inevitable for children with persistent wheezing in China. All the presently included children with persistent wheezing had varying degrees of antibiotic use. The rate of antibiotic treatments was similar between the persistent wheezing children with and without recurrence. Since bronchoscopy was performed via the nose, BAL samples would inevitably be contaminated. However, we followed standard bronchoscopy procedure for each patient to minimize the risk of contamination.

## Conclusion

The overabundances of *Elizabethkingia* and *Rothia* in the BAL of children with PW suggest that the imbalance of airway microbiota could be associated with the development of wheezing. Moreover, this early airway microbial alteration could also be correlated with wheezing recurrence later in life. In addition, the potential role of early airway microbial alteration in pathogenesis of children wheezing will need further investigation.

## Data Availability

The datasets used and/or analyzed during the current study are available from the corresponding author on reasonable request.

## Abbreviations

BAL: Bronchoalveolar lavage
OTU: Operational taxonomic units
PW: Persistent wheezing
QIIME: Quantitative insights into microbial ecology
rRNA: Ribosomal RNA
